# Prognostic factors of oral squamous cell carcinoma: the importance of recurrence and pTNM stage

**DOI:** 10.1186/s40902-024-00410-3

**Published:** 2024-03-04

**Authors:** Min Jae Kim, Kang-Min Ahn

**Affiliations:** Department of Oral and Maxillofacial Surgery, Asan Medical Center, College of Medicine, University of Ulsan, Seoul, South Korea

**Keywords:** Oral squamous cell carcinoma, Survival, Prognostic factor, Recurrence, Neoplasm staging

## Abstract

**Background:**

Oral squamous cell carcinoma has a poor prognosis. Therefore, prognostic factors are important to increase the survival rate. This study assessed the survival rate and the prognostic factors for survival of patients with oral squamous cell carcinoma.

**Method:**

This study included 168 patients who underwent surgery for oral squamous cell carcinoma between January 2006 and December 2021. The survival rate was analyzed with overall survival and disease-specific survival. The patient’s age, sex, pTNM stage, primary sites (lip, tongue, mouth of floor, mandibular gingiva, maxillary gingiva, mandibular vestibule, maxillary vestibule, retromolar trigone, palate, buccal mucosa, primary intra-osseous site), smoking and alcohol drinking habits, depth of invasion, perineural and lymphovascular invasion, cell differentiation, and postoperative radiotherapy were evaluated to analyze risk factors. Kaplan–Meier methods were used to estimate the survival rates. Cox regression methods were used to investigate the main independent predictors of survival in univariable and multivariable analysis.

**Results:**

Sixty-eight patients died of oral squamous cell carcinoma during follow-up periods. Their overall survival for 5 years was 51.2%, and the disease-specific survival was 59.2%. In univariable analysis, seven factors which are neck metastasis, depth of invasion, cell differentiation, lymphovascular invasion, postoperative radiotherapy, pTNM stage, and recurrence were significantly associated with survival. In multivariable analysis, pTNM stage and recurrence were significantly associated with survival.

**Conclusion:**

In patients with oral squamous cell carcinoma, pTNM stage and recurrence were significant prognostic factors. Neck metastasis, depth of invasion, cell differentiation, lymphovascular invasion, and postoperative radiotherapy were also prognostic factors. These factors serve as markers for obtaining prognosis information in oral squamous cell carcinoma.

## Background

Five percent of all tumors occur in the head and neck, with approximately half of those occurring specifically in the oral cavity. Of the 615,000 new cases of head and neck tumors reported worldwide in the 2000s, 300,000 were primary oral squamous cell carcinoma [1]. According to domestic research, approximately 1.5% of the total cancer patients, which is about 1000 patients, are diagnosed with oral squamous cell carcinoma (OSCC) in the oral and maxillofacial region annually [2, 3]. According to statistics from the National Cancer Information Center, 839 cases of oral cancer in South Korea were reported, in 2020. The survival rate of OSCC has been extensively studied by various institutions, resulting in varying rates ranging from 47 to 71%, as the 5-year cumulative overall survival rate among surgically treated patients [4–8]. Furthermore, there has been a slightly upward trend in survival rates over the past two decades, coinciding with advancements in medical technology. Despite significant efforts in recent years, the 5-year survival rate of OSCC patients remains about 60% due to tumor metastasis and subsequent recurrence [9].

The survival of the patient was believed to be influenced by various factors: the patient, the surgeon, and the tumor. It is crucial to establish how these prognostic markers interact and affect the patient’s prognosis. The prognostic markers would be valuable in accurately assessing the biological aggressiveness of these tumors, and enable the development of tailored treatment plans for each specific tumor. However, among these prognostic makers, some markers are challenging to evaluate. Particularly, the surgeon factor presents a greater challenge in this regard. Surgical experience and a well-coordinated team approach involving a medical oncologist and radiotherapist are also crucial factors for achieving successful outcomes. However, assessing the proficiency of the surgeon and the medical oncologist and the radiotherapist can be challenging [6, 8]. Nonetheless, the most well-known evaluable critical factors associated with survival in OSCC include neck metastasis, depth of invasion, and tumor thickness, pTNM stage [4, 8, 10–17]. It is well acknowledged that the presence of neck metastasis is the most crucial prognostic factor in OSCC, resulting in a 50% reduction in patient survival for those with neck metastasis [13, 14]. The neck metastasis is an indicator of an OSCC’s ability to metastasize. Therefore, managing the regional lymphatic is a fundamental consideration in the treatment of an OSCC. Depth of invasion and tumor thickness are also well-recognized for their significant prognostic marker [18, 19]. The pTNM stage, devised by AJCC (American Joint Committee and Cancer), serves as a comprehensive prognostic marker that incorporates the previously mentioned critical factors, including tumor thickness, depth of invasion, and neck metastasis [20]. Furthermore, according to previous studies, it has been established that markers such as age, sex, smoking, drinking habits, sites, perineural invasion, lymphovascular invasion, cell differentiation, postoperative radiotherapy, neck dissection, and recurrence impact the survival of patients [11, 12, 15, 16].

The purpose of this article is to report the overall survival (OS) and disease-specific survival (DSS) outcomes of a consecutive series of patients diagnosed with OSCC between 2006 and 2020 by one surgeon in the Department of Oral and Maxillofacial Surgery at Asan Medical Center (Seoul, Korea). Moreover, we aim to analyze clinically significant prognostic factors which include age, sex, smoking, drinking habits, sites, perineural invasion, lymphovascular invasion, cell differentiation, depth of invasion, postoperative radiotherapy, recurrence, neck dissection, neck metastasis, pTNM stage. Due to the shortage of domestic epidemiological research on the OSCC survival rate and impacting factors, conducted over long-term follow-up, and involving a single surgeon and institution, this research holds particular significance [6, 8, 21–23].

## Methods

This study included 168 patients (112 males and 56 females; 20 ~ 90 years old; mean age 63.4 years) who underwent surgery for OSCC by one experienced surgeon from January 2006 to December 2021. This retrospective study was approved by the institutional review board (IRB) in our hospital (IRB no. S2023-2015–0001) and was conducted in accordance with the Helsinki Declaration of 1975 as revised in 2000.

In this study, the inclusion criteria were OSCC that were primary lesion, single site, and resectable. The exclusion criteria were referred patients after recurrence, referred patients after neoadjuvant chemotherapy, unresectable lesions, not acceptable for general anesthesia, multiple primary OSCC, oral cavity cancer other than OSCC, presence of distant metastasis at initial work-up, and refusal of surgical treatment.

Our department performed radical primary surgery and treated with free flap reconstruction only in cases where primary closure is not feasible. The surgery involves a resection with a safety margin of 1 to 1.5 cm and with a pathologically confirmed margin of 5 mm. If positive neck metastasis was suspected on computed tomography (CT), magnetic resonance imaging (MRI), positron emission tomography (PET), or manual examination, a neck biopsy was confirmed through sono-guided fine needle biopsy. The neck dissection was performed based on advanced results. Additionally, in cases where a free flap was necessary, supraomohyoid neck dissection (SOHND) was performed for the purpose of vessel preparation. During the surgery, multiple frozen biopsies were conducted. If the biopsy result was a close or positive margin, subsequent frozen biopsies were conducted until the attainment of a verified negative margin. After performing the mass excision and confirming the presence of a clear margin, the reconstruction was performed with a microvascular free flap for cases in which achieving primary closure was not feasible. A small soft tissue defect was conducted with the radial forearm flap, and a large soft tissue defect was conducted with the latissimus dorsi flap. In cases involving osseo-cutaneous defects, the fibular-free flap was conducted. The primary role of radiation in oral cavity cancer is in the postoperative setting, with the potential for persistent disease. Patients underwent postoperative radiotherapy if they had positive neck metastasis, extracapsular extension (ECS) of cancer beyond the confines of a node, poor histologic factors (perineural invasion, lymphovascular invasion), and large primary cancers (T3 or T4) [24].

After treatment, the follow-up procedures included CT, MRI, PET/CT at 3- to 6-month intervals. Any cases confirmed as recurrences through imaging or biopsy during follow-up were considered as such. Patient deaths were confirmed based on medical records, and insurance eligibility.

The 5-year cumulative OS and DSS were evaluated. OS was calculated as the survival rate regardless of the cause of death. DSS evaluated the survival rate specifically for cases in which OSCC was the cause of death. This evaluation excluded patients who had recovered their health from OSCC but subsequently died due to other diseases. Kaplan–Meier analysis was used to calculate the 5-year cumulative survival rates for OS and DSS rate.

Age, sex, pTNM stages, primary sites (lip, tongue, mouth of floor, mandibular gingiva, maxillary gingiva, mandibular vestibule, maxillary vestibule, retromolar trigone, palate, buccal mucosa, primary intra-osseous site), smoking and alcohol drinking habits, depth of invasion, perineural and lymphovascular invasion, cell differentiation, postoperative radiotherapy, and recurrence were examined to identify factors predictive for survival. Cox regression techniques were used to investigate the main independent predictors of survival, for the univariable analysis. In univariable analysis, recurrence, pTNM stage, neck metastasis, depth of invasion, cell differentiation, lymphovascular invasion, and postoperative radiotherapy were prognostic factors, significantly associated with DSS. Among these prognostic factors, a multivariable analysis was conducted to identify independent factors. To assess multicollinearity, linear regression analysis was employed in multivariable analysis.

The level of significance was set as *p* < 0.05. Statistical analyses were carried out using the IBM SPSS for Windows (ver. 21.0; IBM Corp., Armonk, NY, USA).

## Results

**Fig. 1:**
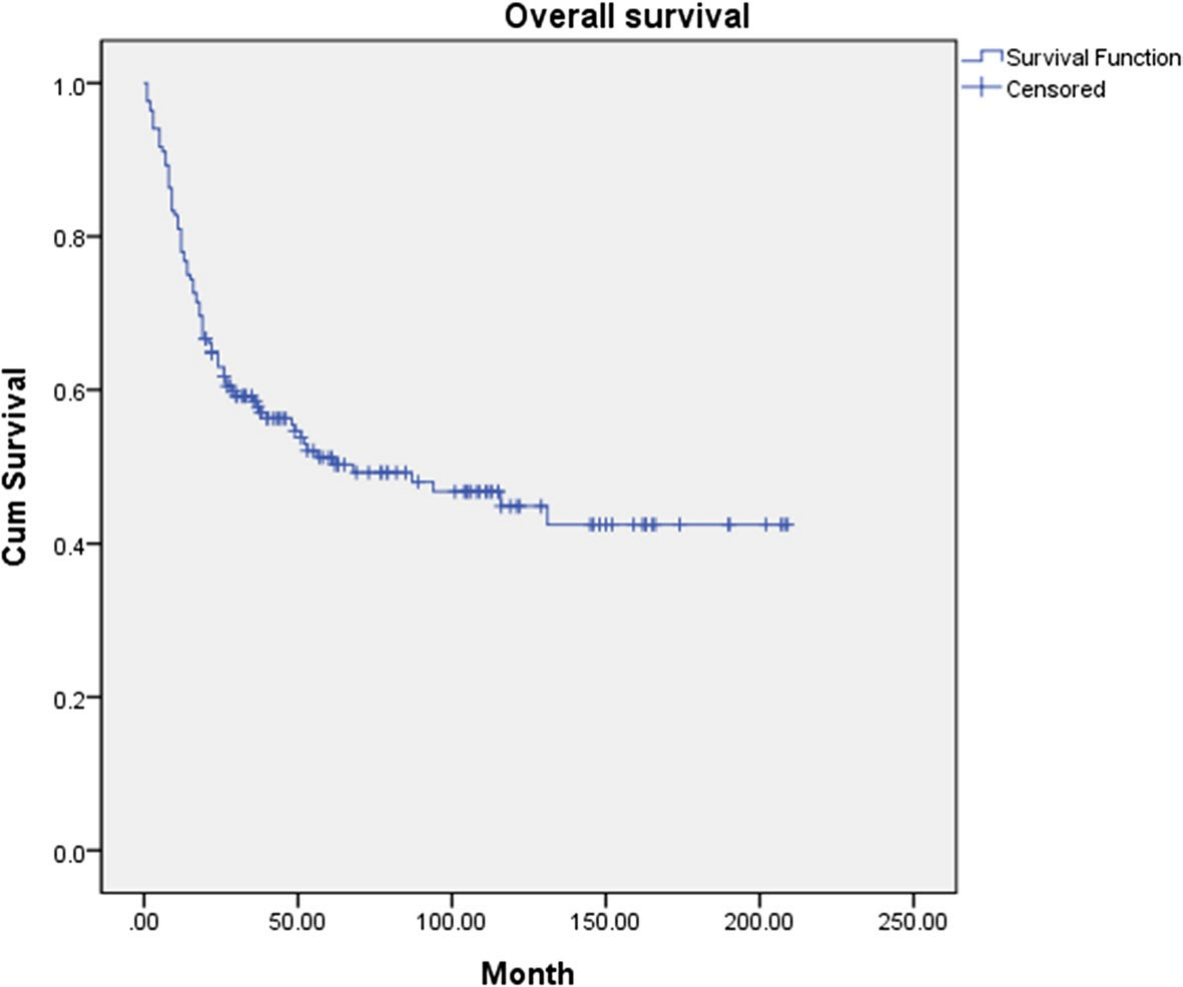
The 5-year cumulative overall survival (OS) of 168 patients with oral squamous cell carcinoma

**Fig. 2:**
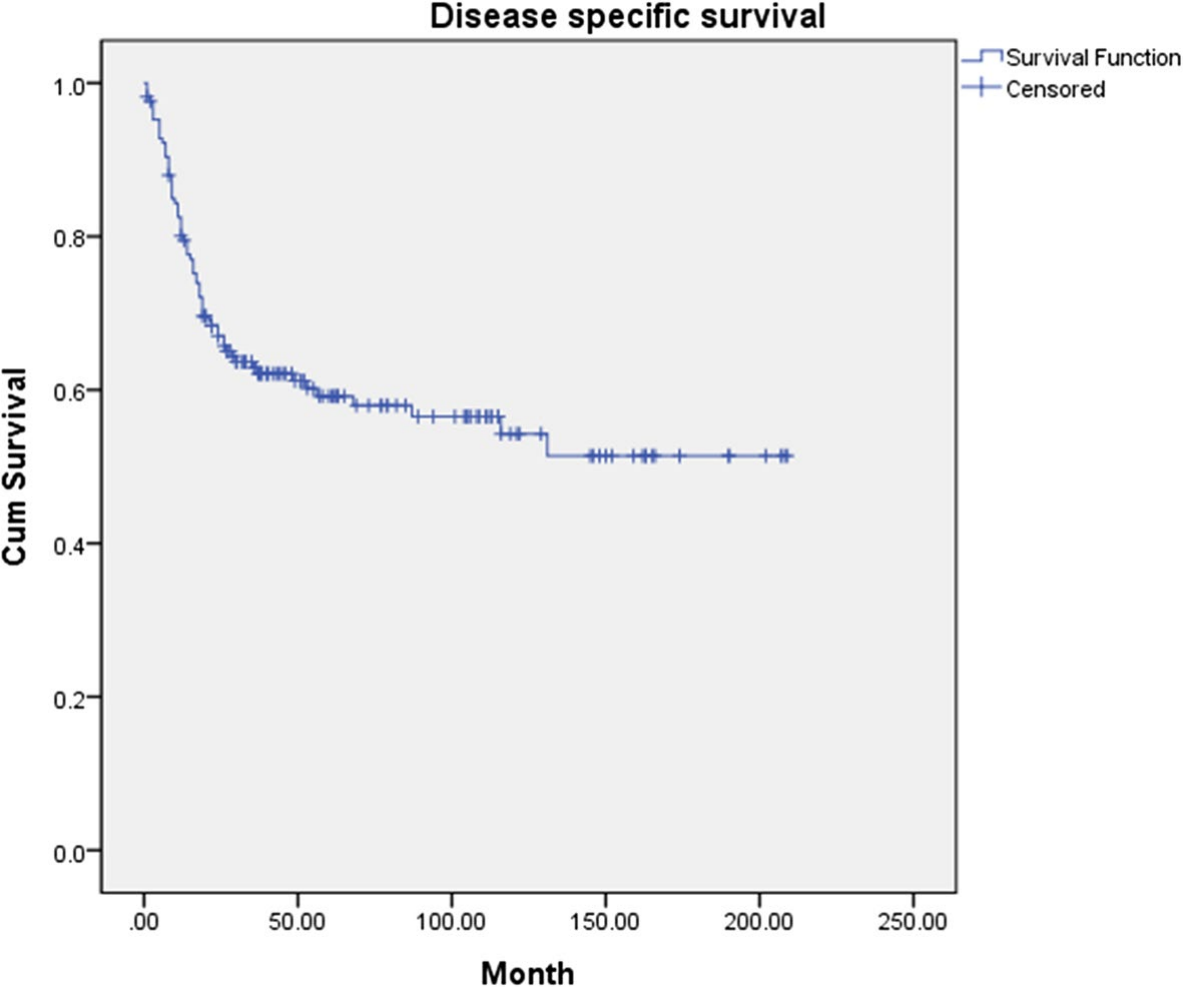
The 5-year cumulative disease-specific survival (DSS) of 168 patients with oral squamous cell carcinoma

A total of 84 patients died during the follow-up period. Among them, 68 patients died of OSCC. The 5-year cumulative survival rates of OS and DSS were 51.2% and 59.2%, respectively (Figs. 1 and 2). The main causes of mortality in OSCC patients were postoperative complications such as aspiration pneumonia due to bleeding and airway obstruction, and general weakness and increased comorbidities due to postoperative cachexia. 16 patients died of other diseases: lung cancer (*n* = 3), gastric cancer (*n* = 1), thyroid cancer (*n* = 1), cerebrovascular disease (*n* = 3), general weakness (*n* = 6), and pneumonia (*n* = 2). The other types of cancer were counted as primary occurrences, rather than metastatic oral squamous cell carcinoma.

Sixty-eight patients died of OSCC. Among the 68 patients’ deaths, 63 patients were related to OSCC recurrence: lung metastasis (*n* = 2), local recurrence (*n* = 42), secondary primary recurrence (*n* = 4), neck recurrence (*n* = 12), and both neck and local recurrence (*n* = 12). Among the 68 patient deaths, 5 were not associated with recurrence: aspiration pneumonia (*n* = 2), comorbidity (chronic obstructive pulmonary disease = 1), unknown (*n* = 2). Two patients, with unknown states, have no recorded follow-up charts as they were treated with radiation therapy at another hospital. But they died 1 month and 3 months after surgery, suggesting that they may have died of post-op complications. In univariable analysis, recurrence, pTNM stage, neck metastasis, depth of invasion, cell differentiation, lymphovascular invasion, and postoperative radiotherapy were significantly associated with DSS (Table 1).

**Table 1:** Univariate analysis of the factors influencing the survival rate of OSCC

Factor	*n* (%)	DSD		DSS (%)	Hazard ratio	*P* value
Sex					1.07	.79
	Male	67	112	59.8		
	Female	33	56	58.9		
Age (year)					1.09	.82
	≥ 50	87	147	59.2		
	< 50	13	21	61.9		
Smoking					0.855	.52
	Yes	48	78	61.5		
	No	52	90	57.8		
Alcohol					0.787	.35
	Non or light	57	101	56.4		
	Heavy	43	67	64.2		
TNM stage					12.85	< .001
	Stage I, II	59	64	92.2		
	Stage III, IV	41	104	39.4		
NM					3.78	< .001
	Positive	10	24	41.7		
	Negative	90	144	62.5		
DOI					3.86	< .001
	< 5	44	54	81.5		
	≥ 5	56	114	49.1		
PNI					1.393	.141
	Yes	8	17	47.1		
	No	92	151	60.9		
LVI					2.07	.016
	Yes	10	24	41.7		
	No	90	144	62.5		
ND					3	.537
	Yes	58	110	40.8		
	No	71	58	73.2		
RT					2.41	< .001
	Yes	32	73	43.8		
	No	68	95	71.6		
Site						.179
	Lip	1	2	50		
	Tongue	21	38	55.3		
	FOM	12	15	80		
	Mn gingiva	21	39	53.8		
	Mx gingiva	12	21	57.14		
	Mn vestibule	1	2	50		
	Mx vestibule	0	0			
	RMT	7	11	63.6		
	Hard palate	0	1	0		
	Soft palate	1	3	33.3		
	BM	21	27	77.8		
	PIOS	3	9	33.3		
Recurrence					14.07	< .001
	Yes	20	81	24.7		
	No	80	87	92		
Differentiation						.004
	Well	51	77	66.2		
	Moderate	49	87	56.3	1.53	
	Poor	0	4	0	8.71	

*DSD* disease-specific death, *DSS* disease-specific survival, *NM* neck metastasis, *DOI* depth of invasion, *PNI* perineural invasion, *LVI* lymphovascular invasion, *ND* neck dissection, *RT* radiotherapy, *FOM* floor of mouth, *Mn.* mandible, *Mx*. maxilla, *RMT* retromolar trigone, *BM* buccal mucosa, *PIOS* primary intraosseous squamous cell carcinoma)

**Fig. 3:**
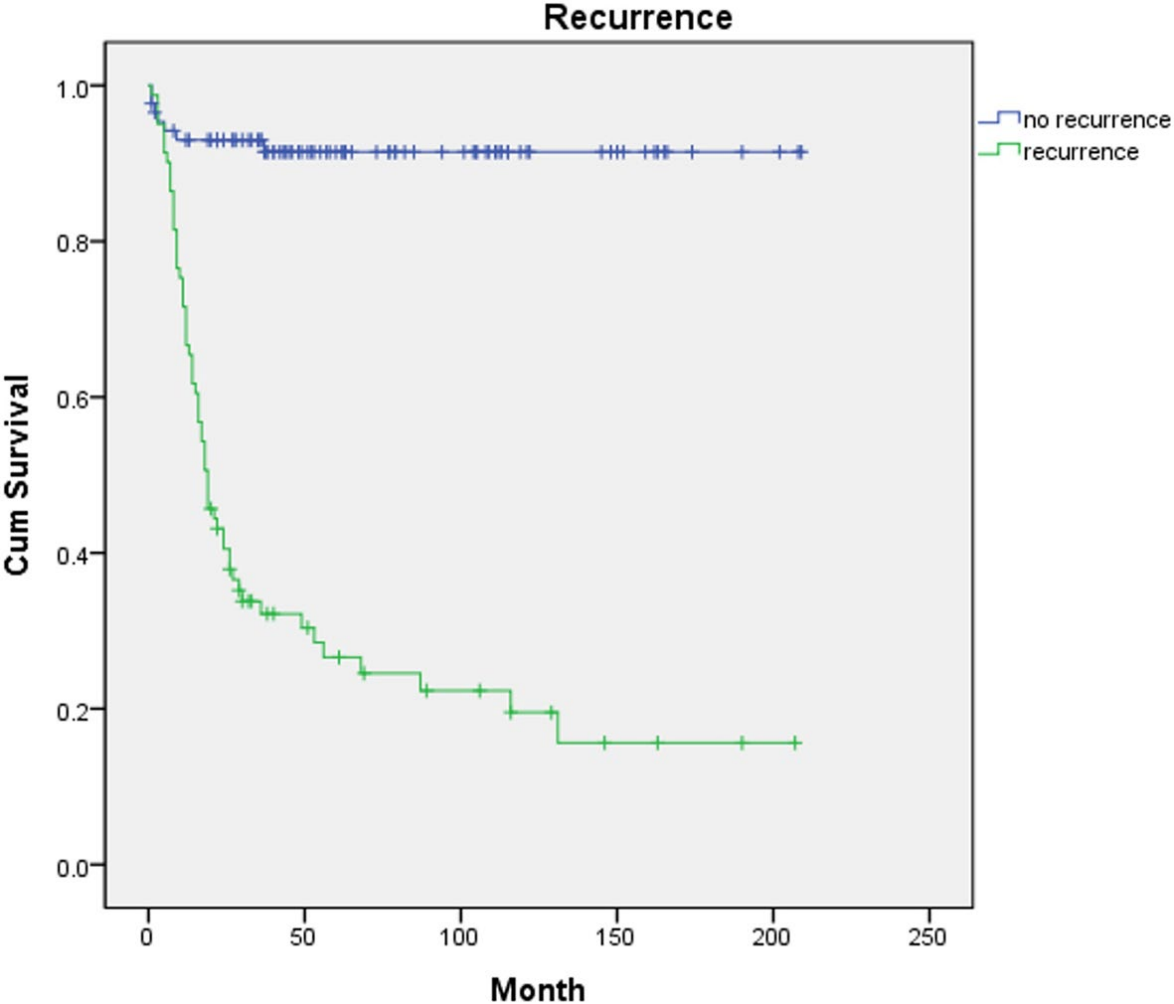
Disease-specific survival for 168 patients with oral squamous cell carcinoma by recurrence

**Fig. 4:**
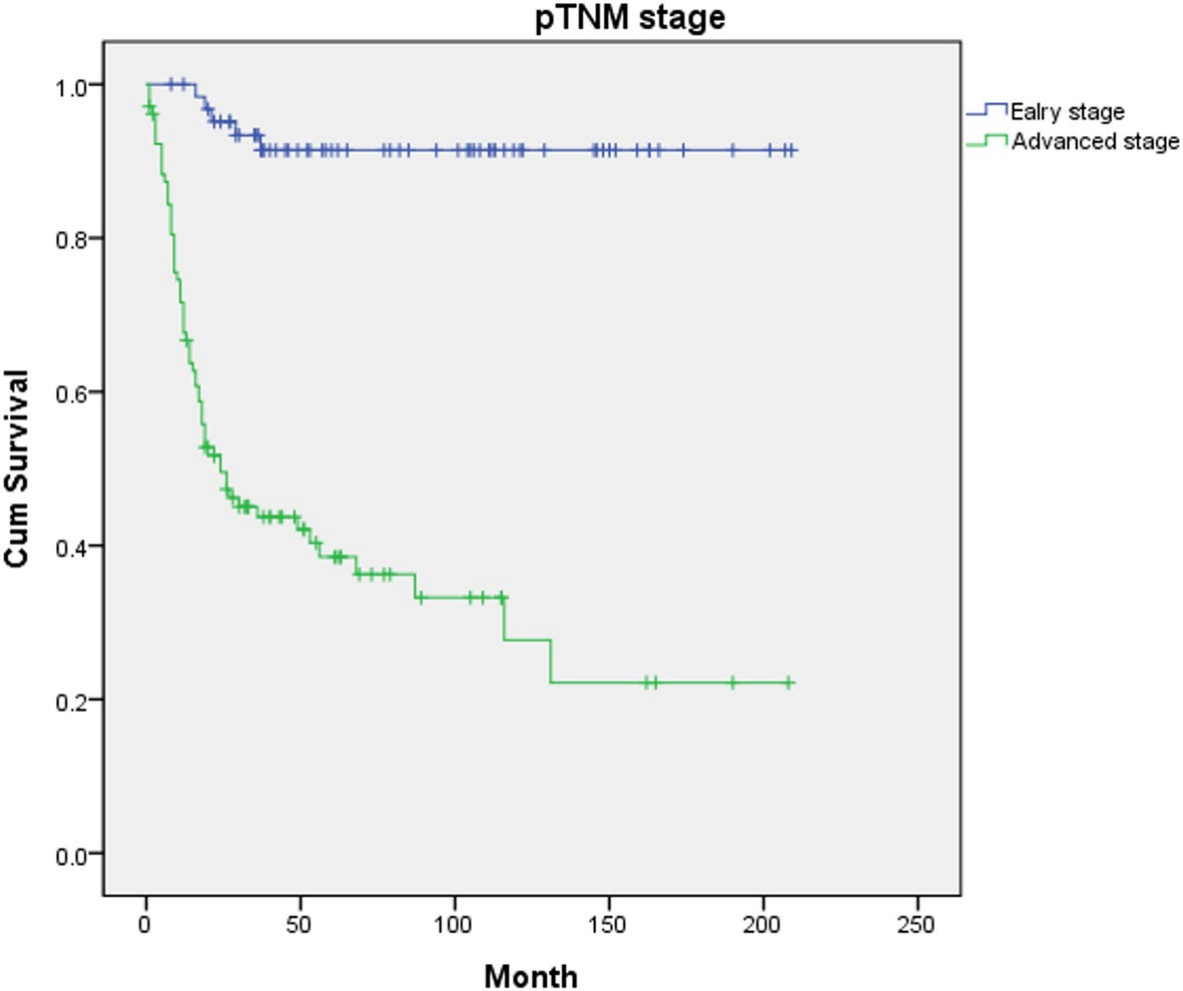
Disease-specific survival for 168 patients with oral squamous cell carcinoma by pTNM stage

Recurrence was found in 81 patients, including local recurrence, neck recurrence, secondary primary recurrence, and lung metastasis. Recurrence was significantly associated with DSS (*p* < 0.001, hazard ratio = 14.07) (Fig. 3). Twenty-seven patients had pTNM stage I disease, followed by stage II (*n* = 37), stage III (*n* = 8), and stage IV (*n* = 96). In this study, the patient group was categorized into early stages, including stage I and II, and advanced stages, including stages III and IV. In the early stages, the DSS was 92.2%, while in the advanced stage, the DSS was 39.4%. In Cox regression, pTNM was significantly associated with DSS (*p* < 0.001, hazard ratio = 12.48) (Fig. 4). Neck node metastasis was found in 24 patients. The DSS was 41.7% in neck node metastasis patients. In Cox regression, neck metastasis also was significantly associated with DSS (*p* < 0.001, hazard ratio = 3.78). Seventy-one patients (42.2%) underwent neck dissection and 73 patients (44.0%) underwent radiotherapy. Neck dissection was not significantly associated with DSS. But radiotherapy was significantly associated with DSS (*p* < 0.001, hazard ratio = 2.41). Through histopathology examination, differentiation, depth of invasion, and lymphovascular invasion were significantly associated with DSS (*p* < 0.001, *p* < 0.0001 and hazard ratio = 3.86, and *p* = 0.012 and hazard ratio = 2.07 retrospectively). The perineural invasion was not significantly associated with DSS. The primary site was not significantly associated with DSS. Mandibular gingiva (*n* = 39) was the most common site for primary lesion sites, followed by tongue (*n* = 38), buccal mucosa (*n* = 27), maxillary gingiva (*n* = 21), floor of mouth (*n* = 15), retromolar trigone (*n* = 11), intraosseous site (*n* = 9), soft palate (*n* = 3), lip (*n* = 2), mandibular vestibule (*n* = 2), and hard palate (*n* = 1).

In multivariable analysis, recurrence and pTNM stage were significantly associated with DSS in Table 2 (*p* < 0.001 and hazard ratio = 8.09 and *p* = 0.002 and hazard ratio = 5.08). In terms of multicollinearity, all Pearson correlations were lower than 0.9, validating the suitability of the factors for multivariable analysis (Table 3).

**Table 2:** Multivariate analysis of the prognostic factors influencing the survival rate of OSCC

Variable		Hazard ratio	*P* value
pTNM stage		5.080	.002
Neck metastasis		1.250	.431
Depth of invasion		1.160	.692
Lymphovascular invasion		1.460	.244
Radiotherapy		.810	.439
Differentiation	Well		.145
	Moderate	1.260	.382
	Poor	2.990	.052
Recurrence		8.090	.000

**Table 3:** Linear regression analysis for the prognostic factors influencing the survival rate of OSCC

Factor		Recurrence	pTNM stage	LNM	DOI	Differentiation	RT	LVI
Recurrence	Pearson Correlation	1	.463^a^	.373^a^	.307^a^	.162^b^	.356^a^	.117
	Sig. (2-tailed)		.000	.000	.000	.036	.000	.132
pTNM stage	Pearson Correlation	.463^a^	1	.518^a^	.562^a^	.123	.366^a^	.145
	Sig. (2-tailed)	.000		.000	.000	.114	.000	.061
NM	Pearson Correlation	.373^a^	.518^a^	1	.205^a^	.067	.361^a^	.211^a^
	Sig. (2-tailed)	.000	.000		.008	.390	.000	.006
DOI	Pearson Correlation	.307^a^	.562^a^	.205^a^	1	.107	.243^a^	.099
	Sig. (2-tailed)	.000	.000	.008		.166	.001	.202
Differentiation	Pearson Correlation	.162^b^	.123	.067	.107	1	.021	.159^b^
	Sig. (2-tailed)	.036	.114	.390	.166		.791	.039
RT	Pearson Correlation	.356^a^	.366^a^	.361^a^	.243^a^	.021	1	.123
	Sig. (2-tailed)	.000	.000	.000	.001	.791		.114
LVI	Pearson Correlation	.117	.145	.211^a^	.099	.159^b^	.123	1
	Sig. (2-tailed)	.132	.061	.006	.202	.039	.114	

*NM* neck metastasis, *DOI* depth of invasion, *LVI* lymphovascular invasion, *RT* radiotherapy

^a^Correlation is significant at the 0.01 level (2-tailed)

^b^Correlation is significant at the 0.05 level (2-tailed)

## Discussion

The study was planned in 2006, and there were no patients dropped out. In several recent studies, the 5-year survival rate was 47 to 71% [4–8]. However, most institutions showed survival rates lower than 60% [9]. In comparison, the 5-year cumulative OS and DSS in our study were 51.2% and 59.2%, retrospectively, which were quite favorable comparable results.

In multivariable analysis, the independent predictors statistically selected for DSS were recurrence and pTNM stage (*p* < 0.001 and hazard ratio = 8.09 and *p* = 0.002 and hazard ratio = 5.08) (Table 2). Specifically, considering that the most of patients who died of OSCC had recurrence (95.6%), recurrence is a significant prognostic factor. Even if the tumor recurs and the patient undergoes salvage surgery, a recurrent tumor grows deeper within the primary site or in the cervical region, and it often positions itself in a location that is hard to excise. These poor situations result in the difficulties of achieving complete resection due to the surrounding anatomical structures [6]. These difficulties result in an increased incidence of postoperative complications, such as aspiration pneumonia due to bleeding and airway obstruction. After salvage surgery, or if salvage surgery is not feasible, patients die due to general weakness and increased comorbidities resulting from malnutrition and cachexia. Additionally, patients died due to pneumonia induced by respiratory depression resulting from the use of opioids for pain management. However, recurrence is a result that occurs after surgery, it is difficult to predict recurrence in advance. In contrast, the pTNM stage can be predicted from the cTNM stage, and additionally, surgery, radiation therapy, and chemotherapy can be administered accordingly. Therefore, the pTNM stage is also an important prognostic factor in planning surgery and management. The pTNM stage has correlations with recurrence (*r* = 4.463, *p* < 0.001) (Table 3), and is a comprehensive factor that includes tumor size, invasion, and neck metastasis. these factors are more likely to contribute to the difficulties of achieving a clear resection margin and the formation of tumor budding and tumor cell nests. These increased postoperative complications, patient comorbidities, and tumor recurrence, ultimately result in a decreased patient’s survival rate.

In univariable analysis, neck metastasis, depth of invasion, cell differentiation, lymphovascular invasion, and postoperative radiotherapy were also statistically significant prognostic factors (Table 1). Particularly, neck metastasis and depth of invasion were well-known prognostic factors in the previous study [13, 14, 17, 25–27], and were included in the criteria for determining pTNM stage. This demonstrates the statistically significant correlation with the pTNM stage. (neck metastasis *r* = 0.518, *p* < 0.001, depth of invasion: *r* = 0.562, *p* < 0.001) (Table 3). The neck node metastasis and depth of invasion are indicators of an OSCC’s ability to metastasize, invasion section margin, and the formation of tumor budding and tumor cell nests [28]. These increased tumor recurrence, ultimately result in a decreased patient survival rate. This demonstrates the statistically significant correlation with recurrence (neck metastasis *r* = 0.373, *p* < 0.001, depth of invasion: *r* = 0.307, *p* < 0.001). Cell differentiation was also a well-known prognostic factor. However, it was not included in the criteria for determining TNM stage [29, 30]. This demonstrates no statistically significant correlation with the pTNM stage (*p* = 0.114). But, poorly differentiated tumors were associated with a recurrence (*r* = 0.162, *p* = 0.036), resulting in a decreased survival rate. Postoperative radiotherapy was also found to have statistical significance in relation to DSS. The indications for postoperative radiotherapy: positive neck metastasis, poor histologic factors, and large primary cancers are already crucial prognostic factors for survival rate [24]. This demonstrates the statistically significant correlation with recurrence, pTNM stage, neck metastasis, depth of invasion, and differentiation. These correlations result in a decreased survival rate. Lymphovascular invasion was also found to have statistical significance in relation to DSS. It would seem reasonable that the presence of the lymphovascular invasion at the primary site would predict neck metastasis since the invasion of lymphatic is the initial step in forming a metastasis [31, 32]. Considering neck metastasis as a critical prognostic factor, these correlations result in a decreased survival rate. This demonstrates the statistically significant correlation with neck metastasis in Table 3 (neck metastasis *r* = 0.211, *p* = 0.006).

In this study, patient factors such as age, sex, smoking status, and alcohol habits were found not to have a statistically significant impact on survival. Young age (< 50) represented higher DSS than older age (≥ 50), but there were no statistically significant. Similar studies focusing on age groups emphasized results where older patients had a lower survival rate compared to younger patients [33, 34]. And according to previous research, smoking and alcohol habits are recognized as primary risk factors for OSCC [35]. But it’s notably observed that the most of smokers and drinkers are men, while about one-third of the women (male:female = 112:56) in this study do almost not smoke or drink alcohol. Women, in previous studies, have been reported to have lower survival rates compared to men, also in the present study [27, 36] The patient factors, that exhibited conflicting and complex interrelationships, proved challenging to establish statistical significance. But above all, there are stronger factors (recurrence, pTNM stages etc.) that affect survival, and make the other factors less apparent in the statistical significance. There were controversies regarding whether surgical sites are a prognostic factor for survival or not, as cervical metastasis and bone and muscle invasion may manifest differently depending on the sites [21, 22, 37, 38]. This can be overcome by surgically removing the tumor and lymph nodes with a sufficient margin. therefore, it did not have a significant impact on the survival rate in this study. In this study, neck dissection included elective neck dissection (*n* = 20) and therapeutic neck dissection (*n* = 90). Among the total cN0 cases (*n* = 78), lymph node recurrence (*n* = 11) was 14.1%, and total lymph node recurrence (*n* = 26) was 15.5%. These were comparable to findings in previous studies [39, 40]. Therefore, it can be seen that all types of neck dissection were conducted well with almost no neck failure. However, neck dissection did not yield statistically significant results in this study. Since this study was not designed to randomize patients and evaluate differences in survival based on neck dissection or its absence, and elective neck dissection or therapeutic neck dissection, it would be hard to evaluate whether neck dissection and elective neck dissection or therapeutic neck dissection has a relation to survival or not.

The novelty of this study lies in the fact that it was conducted by a single surgeon within a single institution. In this study, we adjusted factors related to the surgeon, oncologist, and radiation oncologist that may affect the survival of patients with oral squamous cell carcinoma (OSCC). Additionally, our results were consistent with those of previous studies [6, 9, 13, 19, 20]. However, the limitations of the study include a relatively modest sample size and a retrospective study design. It will be necessary to address this limitation by supplementing it with more accumulated clinical case reports in the future.

## Conclusion

Oral squamous cell carcinoma (OSCC) exhibits a poor prognosis, with a 51.2% overall survival (OS) rate and a 59.2% disease-specific survival (DSS) rate. Multivariable analysis identified pTNM stage and recurrence as independent and direct prognostic factors. While recurrence significantly impacts survival, predicting it perioperatively remains challenging. Conversely, the pTNM stage was reaffirmed as a predictive and independent prognostic factor. In univariable analysis, neck metastasis, depth of invasion, cell differentiation, lymphovascular invasion, and postoperative radiotherapy were also identified as prognostic factors. Therefore, representing these findings perioperatively necessitates close observation and intensive care to enhance the patient’s chance of survival.

## Data Availability

Not applicable.

## Abbreviations

OSCC: Oral squamous cell carcinoma
OS: Overall survival
DSS: Disease-specific survival
AJCC: American Joint Committee and Cancer
CT: Computed tomography
MRI: Magnetic resonance imaging
PET: Positron emission tomography
SOHND: Supra-omohyoid neck dissection
ECS: Extracapsular extension
DSD: Disease-specific death
NM: Neck metastasis
DOI: Depth of invasion
PNI: Perineural invasion
LVI: Lymphovascular invasion
ND: Neck dissection
RT: Radiotherapy
FOM: Floor of mouth
Mn.: Mandible
Mx.: Maxilla
RMT: Retromolar trigone
BM: Buccal mucosa
PIOS: Primary intraosseous squamous cell carcinoma
